# Recent Advances for the Detection of Ochratoxin A

**DOI:** 10.3390/toxins7124882

**Published:** 2015-12-04

**Authors:** Tai Hwan Ha

**Affiliations:** 1 BioNanotechnology Research Centre, Korea Research Institute of Bioscience and Biotechnology (KRIBB), 125 Gwahak-ro, Yuseong-gu, Daejeon 34141, Korea; taihwan@kribb.re.kr; Tel.: +82-42-860-4272; Fax: +82-42-879-8596; 2 Nanobiotechnology (Major), Korea University of Science & Technology, 125 Gwahak-ro, Yuseong-gu, Daejeon 34141, Korea

**Keywords:** mycotoxins, ochratoxin A, aptamers, amplified detection

## Abstract

Ochratoxin A (OTA) is one of the mycotoxins secreted by *Aspergillus* and *Penicillium* that can easily colonize various grains like coffee, peanut, rice, and maize. Since OTA is a chemically stable compound that can endure the physicochemical conditions of modern food processing, additional research efforts have been devoted to develop sensitive and cost-effective surveillance solutions. Although traditional chromatographic and immunoassays appear to be mature enough to attain sensitivity up to the regulation levels, alternative detection schemes are still being enthusiastically pursued in an attempt to meet the requirements of rapid and cost-effective detections. Herein, this review presents recent progresses in OTA detections with minimal instrumental usage, which have been facilitated by the development of OTA aptamers and by the innovations in functional nanomaterials. In addition to the introduction of aptamer-based OTA detection techniques, OTA-specific detection principles are also presented, which exclusively take advantage of the unique chemical structure and related physicochemical characteristics.

## 1. Introduction

**Figure 1:**
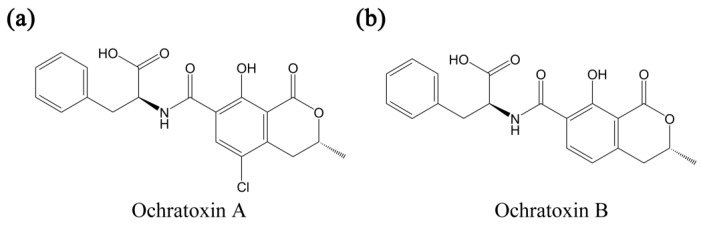
Chemical structures of ochratoxin A (**a**) and ochratoxin B (**b**).

Ochratoxins are toxic metabolites that are secreted by fungi species such as aspergillus and penicillium [1,2,3], and constitute a main group among more than 400 mycotoxins with diverse chemical structures and toxic effects. It has been known that those mycotoxins are responsible for several diseases and regulatory authorities only became aware of their threat toward public health after the 1960s. The host fungi of ochratoxins cannot only colonize various grains like coffee, peanut, and rice, but also can contaminate commercialized food systems (secondary products of infected grains) like wine, bread, and meat products [4,5,6,7,8,9]. Furthermore, the most harmful, ochratoxin A (OTA), can survive eradication of host fungi and exhibits an unusually long half-life of 35 days in the human body [10]. OTA has nephrotoxic, immunotoxic, and teratogenic effects, and was classified as a possible carcinogen by the International Agency for Research on Cancer (IARC) [11]. Also, it attracted research attention as one of the endocrine disrupters or a reproductive toxicant that deteriorate male or female reproductive health [12]. The harmful effects of OTA are postulated to be related to its structural origin, the amide linkage of phenylalanine with the 7-carboxylic group of the dihydroisocoumarin moiety (see Figure 1); OTA appears to compete with natural phenylalanine in the protein synthesis involving phenylalanine-tRNA synthetase. It has also been reported that OTA affects the inhibition/activation of enzymes in protein synthesis and apoptosis [13,14], immunosuppression, reduction of immune organ [15], depression of antibody responses, alteration in immune cell activities [16], and modulation of cytokine production [17].

Regulations that detail the tolerances of OTA along with experimental protocols have been prepared for foodstuffs and animal feedstuffs [18,19,20]. In the European Union, regulatory limits of OTA levels in food systems are now strictly laid down; the upper concentration in cereals was set to 5 ppb, while 2 ppb and 5 ppb was set for wine (or grape juice) and to coffee products, respectively [19,21,22]. In a case study performed in the Czech republic, the actual OTA levels in foodstuffs were determined [23], and the detailed relations between the dietary intake of OTA and its concentrations in human samples (like milk, blood, urine, and kidney tissue) were discussed and compared with data from other countries [24]. In order to meet these detection limits with a variety of background matrixes, conventional detection methods have been applied such as ELISA [25], thin layer chromatography [26], and HPLC [27]. Simultaneously, simple and on-site detection methods have been continuously developed in parallel, which are mostly propelled by development of specific DNA aptamers and innovative functional nanomaterials.

## 2. Conventional Detection Methods for OTA

Since most mycotoxins are chemically stable, OTA tends to survive the eradication of host fungi such as cooking in boiling water, baking bread, or long-time storage at ambient condition. Therefore, the absence of visible fungi mold in grains is not indicative of being free from the contamination [28]. These characteristics make it crucial to avoid the formation of OTA itself as a precautionary measure, which is not always possible in the real world. Second, due to diverse characteristics of mycotoxins in terms of their physicochemical properties, it seems almost impossible to prepare one golden technique to cover all mycotoxins threatening public health. Therefore, the best applications for OTA cannot apply to other mycotoxins automatically. Third, as OTA presents toxicity in a very low concentration, analyzing this molecule generally needs very sensitive instruments with well-trained handlers, which are practical challenges for on-site OTA assays of various food samples. For future applications, simple detection methods with non-scientific personnel will be enthusiastically pursued, which should provide a route for rapid and on-site detections, preventing contaminated grains from spreading to secondary products or to consumers. Fourth, for the development of practical and efficient OTA monitoring, the detection scheme should be robust to diverse matrix materials, sensitive up to regulation levels, and flexible to the sample status because a variety of samples need to be examined. Especially for field monitoring, rapid and portable sensor systems might be desirable. In the next sections, traditional but powerful detection methods like HPLC and immunoassays are surveyed with a brief estimation of sensitivities and samples preparations.

### 2.1. Chromatographic and Immunoassays of OTA

In order to examine the OTA contents in various samples, a myriad of investigations has been performed hitherto, which can be roughly categorized into two criteria, instrumental analyses and immunoassays. Instrumental analyses generally boast of good accuracy and reproducibility, which include high-performance liquid chromatography (HPLC), liquid chromatography tandem mass spectrometry (LC-MS/MS); the limit of detection (LOD) being as low as 0.05 μg/kg [29], and 0.01 ng/mL [30], respectively. Despite their supreme sensitivities and versatility in many real samples, those methods are time-consuming and usually need well-trained handlers. However, it is also noteworthy that the LC-MS/MS technique has in recent years emerged as the method of choice for multi-residue analyses of mycotoxins [31] or pesticides [32] remaining in our surroundings, since the quick, easy, cheap, effective, rugged, and safe (QuEChERS) method was introduced for sample preparation [33,34]. The QuEChERS protocol substantially alleviated the errors associated with different matrix materials [35] and different physicochemical properties of mycotoxins [36] so that more than 30 mycotoxins could be analyzed simultaneously [31].

Recently, affinity probe capillary electrophoresis (APCE) assay, which combines the separation power of CE and ligand specificity of biomolecules, has emerged as a powerful tool for small molecule assay. In one advanced application, OTA could be selectively discerned in the presence of other targets like adenosine and tyrosinamide by applying their corresponding aptamers as crucial signal tracers to sample solution [37]. The new approach took advantage of resisting aptamers with their target molecules toward phosphodiesterase I enzyme (PDE I) that catalyze the removal of nucleotide from DNA in the 3′ to 5′ direction. Therefore, fluorescein tagged OTA aptamer (F-Apt-O) can resist the degradation in the presence of the target and show a distinct band while disappearing in the absence of OTA. Consequently, the OTA aptamer protected against PDE I activity by the target complexation was proportional to the OTA concentration. The LOD for OTA was measured to be 140 nM and provided a linear range up to 700 nM. In another CE application for OTA detection, the OTA aptamer was utilized for constructing a miniaturized pre-concentration unit in a monolithic capillary support [38]. The OTA aptamers were first immobilized on the initial 1.5 cm region of a 30 cm capillary column to provide a mini-LC segment by UV photografting of a thiol-modified aptamer onto a vinyl spacer on the column. Under optimized condition, OTA was successfully pre-concentrated and quantified down to 0.1 pg (percolation of 2.65 μL of a 40 ng/L OTA solution) with almost quantitative recovery rate (93 ± 2%).

On the other hand, immunoassays like enzyme-linked immunosorbent assay (ELISA) have been employed to accomplish the analyses within a relatively short time [39]. In a chemiluminescent ELISA study with monoclonal antibody, the LOD toward OTA in spiked soybean samples was measured to be 0.08 ng/mL [40,41]. In an attempt to improve the performance of ELISA, a new fusion protein was produced that linked camel-derived nanobodies with reporting alkaline phosphatase (Nb-AP), which circumvent the difficulties in chemical conjugation of antibodies to HRP in the ordinary ELISA scheme. Compared to single chain fragment of variable region (scFv) that had been usually applied in this field, nanobodies have advantages of a smaller molecular weight (~15,000) and high expression level in the microbial system, while maintaining high selectivity toward targets [42]. Fluorescence polarization immunoassay (FPIA) seems to be another branch of immunoassay, encompassing both regions of the previous classification: an instrumental analysis using OTA-specific antibodies. An analysis with FPIA is relatively fast and a LOD of 0.7 ng/mL was achieved in a red wine sample [43]. For the on-site detection of OTA, several immunochromatographic assays have been attempted with results showing relatively higher detection limits (5–500 ng/mL) [44,45]. A comparative study obtained with ELISA and HPLC showed that ELISA had a tendency to underestimate the OTA content compared to that from HPLC [27]. Nevertheless, ELISA appears an invaluable tool for on-site and rapid detection of OTA. For instance, in an attempt to pinpoint the causes of increasing chronic kidney disease, commercial ELISA kits were thoroughly used to monitor OTA concentrations in commonly obtained food items in Sri Lanka. In contrast to the initial expectation, the levels of OTA found in those food commodities were below the maximum allowance of OTA, suggesting that OTA was not the possible origin of the nephrotoxicity in Sri Lanka [46].

### 2.2. Pretreatment or Enrichment Process for Real Samples

In the course of either instrumental analyses or immunoassay, the enrichment or pre-treatment process of real samples like cereals or their secondary products (such as bread or wine) seems to be a crucial factor both in the accuracy and in the time consumption, and affects the final determinations of OTA in samples of interest. In fact, underestimation and overestimation of OTA contents were observed in wine and wheat samples, respectively, and attributed to the pH dependence of the chemical stability of OTA in the clean-up step and the citrinin interference in the process of immunoaffinity column cleanup [47]. Generally, the extraction method employed to enrich a mycotoxin from complex biomatrixes is strongly dependent on the nature of the mycotoxin. Most analytical methods for assaying OTA adopted specialized extraction methods like liquid-liquid extraction (LLE), in which the different solubility of the toxin between aqueous phase and immiscible organic phase was utilized to extract it from the other matrix materials. For instance, in assaying OTA concentration of pig tissues, liquid-liquid extraction was employed and 74% or 86% of recovery (but with relatively low reproducibility) were reported for kidney and muscle tissue, respectively [48]. In these extractions, solvents such as hexane and cyclohexane are supposed to remove non-polar contaminants such as lipid or cholesterol. However, the procedures are generally time-consuming and dependent on the matrix as well as the toxin itself, so a commercialized solid phase extraction column (*i.e.*, Ochraprep column) was needed for the pretreatment of a pig liver sample [48]. Since OTA is largely categorized into hydrophilic materials compared to matrix materials, reverse phase chromatography was often employed for enrichment or prior clean-up compared to LLE. Using C18 reverse phase column chromatography as a clean-up process from matrix materials, 90% recovery of OTA from sweet wine samples from Spain was reported [49].

In the meantime, as another pretreatment measure, the immunoaffinity column has been an alternative choice in the detection of OTA. In the estimation of OTA contents of 340 wines in Portugal, it was revealed that ethanol and glucose content did not interfere in the clean-up of OTA by immunoaffinity columns [50]. In a separate study for determination of OTA in wine grapes, extraction with hydrogen carbonate and poly(ethylene glycol) (PEG) solution (5% NaHCO_3_ and 1% PEG-8000) was followed by the immunoaffinity clean-up procedure. In the next precision assay using HPLC-FD, the LOD and the limit of quantification (LOQ) were established at 4 and 7 ng/kg, respectively [51]. Compared to the traditional method that involved extraction with acidic methanol, a better performance was observed with a good correlation. As an alternative approach to IA column for OTA, the Giraudi group prepared a hexapeptide library by combinatorial synthesis employing standard solid phase synthesis, and identified a peptide sequence that has a fair binding constant with OTA, K_eq_ = 3.4 × 10^4^ M^−1^ [52]. A stationary phase holding the hexapeptide was used to develop the SPE column for quantification in wine samples, and demonstrated 95% recovery. In a separate trial, a dodecapeptide (NFO_4_) derived from specific regions of human oxidoreductase demonstrated a fair binding affinity toward OTA, and was used successfully either in competitive ELISA assay (LOD 2 ng/mL) [53] or in competitive chemiluminescence assay (LOD 0.5 ng/mL) [54].

## 3. Development of OTA Aptamers and Their Applications

The development of OTA-specific DNA aptamer sheds a light on new detection methods of high sensitivity and accuracy, providing an additional avenue on OTA-binding biorecepters. OTA aptamer of 36 nucleotides (termed as 1.12.2) was first reported in 2008 [55] and several related techniques for the mycotoxin were soon developed which used the original sequence as a core element for the detection [56,57]. Although no specific 3D conformation of the OTA binding aptamer was given, many doublet or triplets of guanine implicated the structure of guanine tetrad as a postulated binding scheme [58]. In a separate trial for OTA-specific aptamer, a different two aptamers were newly obtained in 2010 [59], which were termed H8 and H18, respectively, each comprising 30 nucleotides. The dissociation constants were measured to be 96 nM for H12 and 130 nM for H8, which were substantially improved values compared to the previous one (200 nM for 1.12.2 OTA aptamer). In the structural comparison of two separate aptamers (1.12.2 *vs.* H12 or H8) using “m-fold” program, it was revealed that two conserved sequences were found [59]. The first one was located in the stem region which stabilizes the whole structure due to high GC contents, while the second consensus region was positioned on the single stranded loop.

The use of aptamers instead of antibodies has numerous advantages in many applications [60]. First, since the aptamer selection process known as SELEX is performed *in vitro* condition, the findings and the subsequent affinity evaluation for corresponding aptamers are robust and straightforward. In contrast, the generation of antibodies is strongly dependent on the *in vivo* condition of animals that produce the antibodies. Low immunogenicity or toxicity of some antigens that cause problems in antibody production, do not interfere with the aptamer selection. Second, the targeted antigens can be freely manipulated *in vitro* conditions, and the reproducibility and purity of the selected aptamers can be strictly controlled as they are chemically synthesized. For instance, reporter or functional groups (even antibodies) can be easily attached to the aptamer [61,62]. Third, compared to proteinaceous antibodies, DNA aptamers are chemically stable and easy to modify via simple chemistry during the synthesis. Also, the lack of large hydrophobic cores (often found in proteins) prevents them from aggregating in varying conditions. Therefore, DNA aptamers can tolerate a wider range of pH and temperature that proteins do not. Fourth, relatively small sizes of aptamers lead to high densities of them on targets, which may have improved their targeting (or transporting also) properties in drug delivery applications [63,64,65].

### 3.1. OTA Aptamers in Affinity Columns and Enzyme Linked Assays

Guided by these universal advantages of aptamers, OTA-binding aptamers showed fair potential to replace corresponding antibodies in the field of immunoassay (ELISA), separation column, or nano-biosensors. An excellent review article is already available in the literature [58], introducing various field researches employing OTA aptamers along with explanations on their mechanisms of transducing signals. Because of the fluorescent nature of OTA, chromatographic techniques (mainly HPLC) have been considered as a gold standard as mentioned above. In a similar context, OTA was used to fabricate an aptamer affinity column (AAC) as a pre-treatment or enriching column. The first aptamer (1.12.2) that was conjugated to agarose resin obtained 97% recovery from spiked buffer solutions, and showed the same result with wheat samples as in a certified method [55]. In an improved preparation of AAC, HPLC analyses for naturally contaminated wheat samples with OTA showed an LOD of 0.023 ng/g and an LOQ of 0.077 ng/g; the same AAC column showed recovery rates of 74%–88% for the spiked wheat samples in the range of 0.5–50 ng/g [66]. In a comparative study using IAC and AAC, good correlation between two protocols was revealed in several contaminated wheat samples. The subsequent detections using time-resolved fluorescence spectra of terbium ions in response to OTA showed a recovery rate of 77% for the spiked wheat samples in the range of 2.5–25 ng/g [67]. Instead of polymeric resins like agarose, magnetic nanoparticles (MNP) were also employed as solid support for OTA aptamers. The MNP-aptamer sorbent showed recovery rates of 67%–90% for the different spiked cereal samples in the range of 2.5–50 ng/g [68]. For wine samples that are considered as the second most contaminated food, covalently immobilized OTA aptamers on cyanogen-bromide-activated (CNBr-activated) sepharose showed better performances in recovering OTA [69]; the same OTA binding sepharose beads (using CNBr-activation) showed a recovery rate of 96% for spiked beer samples [70]. As mentioned before, the small size of aptamers and subsequent high density of immobilization on solid supports seems to improve their retention capacity toward OTA, thereby enhancing the LOD of the following analyses [71].

Since the development of OTA aptamer, a variety of detection schemes have been attempted to tackle the drawbacks of conventional assays [59]. Mimicking the traditional ELISA (termed ELAA, Enzyme-Linked Aptamer Assay) was first attempted to use the developed OTA aptamer (H8 and H12). For the indirect competitive assay, OTA was covalently immobilized on the well and OTA aptamers (H8 or H12), conjugated with a catalytic enzyme (e.g., horseradish peroxidase, HRP), were initially bound with the tethered OTA. The addition of OTA detached some of the pre-immobilized H8-HRP (or H12-HRP) and gave signals proportional to the OTA concentration. A closely related competitive detection scheme could be realized on the waveguide sensor, in which the loss of fluorescence could be detected on the surface of the optical fiber by detecting the amount of remaining aptamer [72]. An integrated evanescent wave all-fiber (EWA) biosensing platform was developed for the binding kinetics between the tethered OTA and its aptamer. With a competitive detection, the quantification of OTA over concentration, ranges from 0.73 ng/mL–12.50 ng/mL with a LOD of 0.39 ng/mL. On the other hand, for the direct competition assays, OTA aptamers were permanently immobilized on the surface and OTAs conjugated with HRP were initially bound with the OTA aptamers. The addition of exogenous OTA detached OTA-HRP conjugates in proportion to the OTA concentration in the sample solution. This detection scheme showed an LOD of 1 ng/mL for the wine samples.

### 3.2. OTA Aptasensors with Functional Nanomaterials

As an interdisciplinary study, gold nanoparticles (AuNPs) were employed as a reaction beacon to detect OTA concentration. Usually, short oligonucleotides with 20–50 base pairs have a tendency to interact with citrate-capped gold nanoparticles to give resistance to the salt-triggered aggregation [73]. However, the addition of OTA prevents the aptamers from interacting with AuNPs, thereby promoting aggregation behavior on high salt concentration. The LOD of this technique (20 nM) was relatively higher than other techniques, but had a very short operation time like 5 min. Instead of the salt-triggered aggregation of AuNPs, seeded growth of AuNPs could be another exotic signal transducing principle [74]. In this scheme, aptamer-target interactions modulate the amount of aptamer strands adsorbed on the surface of initial AuNPs via desorption of the aptamer strands upon binding with OTA. Depending on the resulting aptamer coverage, AuNPs grew into morphologically varied nanostructures in the additional reduction condition with hydroxylamine, which gave rise to different colored solutions. For the detection of OTA, 1 nM of LOD was achieved in a red wine sample while cocaine and 17β-estradiol demonstrated 1 nM and 0.2 nM of LODs for spiked synthetic urine and saliva, respectively. More directly, local surface plasmon resonance (LSPR) of gold nanorod (GNR) could be utilized to monitor the content of OTA in sample solution [75]. Since the magnitude of the LSPR wavelength change is dependent on both the location of the analyte and the extent of alteration of the local refractive index, OTA aptamer tethered on GNR could give rise to sufficient LSPR changes on binding with OTA and consecutive conformational changes of the OTA aptamer. Furthermore, it was demonstrated that once used GNR could be regenerated by simply heating in methanol solution and an LOD of 1 nM was observed for the spiked corn samples.

Recently, core-satellite assembly of nanoparticles opened a new avenue for signal transduction platform employing bioconjugated gold nanoparticles [76,77]. In one example, chiral AuNP (core)-AgNP (satellite) assemblies were fabricated and showed that this architecture had intense chiroptical response with the increased number of satellite NPs; AuNPs (d ~35 nm) were functionalized with the aptamer for OTA while its partially complementary strands were employed in decorating the satellite AgNPs (d ~8 nm) [78]. The core-satellite structure was dismantled to give a decrease of the CD signal in proportion to the OTA content as the enhanced chirality of the hybrid assembly decreased upon OTA-aptamer complexation. The LOD according to the CD measurements was calculated to be 0.16 pg/mL for spiked samples, and a plausible recovery rate of 95%–105% was achieved in red wine samples. According to the previous reports, the chiroptical activity of the core-satellite assemblies was related to the plasmon enhancement of chiral DNA and the dissymmetry of the collective system [79].

**Figure 2:**
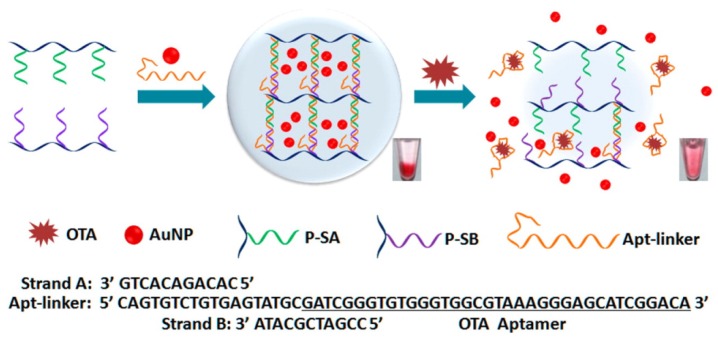
Working principle of the DNA hydrogel encapsulating AuNPs for visual detection of Ochratoxin A (OTA). The formation of OTA-aptamer complex collapses the backbone of the hydrogel network and the preloaded AuNPs in the hydrogel are released, leading to a change of the supernatant from colorless to red, which could be observed by the naked eye (Adapted from Ref. [80], Copyright 2015, American Chemical Society).

As a unique avenue of OTA detection, DNA hydrogel was chosen for the intelligent matrix, in which OTA aptamers were cross-linked with a hydrogel network based on linear polyacrylamide [80]. As shown in Figure 2, the hydrogel network was formed by hybridization between a single DNA strand containing OTA aptamer in part and two partially complementary strands grafting on linear acrylamide chains. In fact, DNA hydrogels with DNA/DNA or DNA/aptamer crosslinks have been exploited for biosensing, bioseparation, and drug release [81,82]. In response to OTA in sample solution, the aptamer in the hydrogel binds with OTA leading to the dissociation of hydrogel, followed by release of the preloaded AuNPs, which can be monitored by the naked eye [80]. For more sensitive detection, Au@Pt core-shell nanoparticles were employed to catalyze the oxidation of H_2_O_2_, thereby generating gaseous O_2_, which induces movement of an ink bar to a distance in a concentration dependent manner (V-Chip system). With an aid of an IAC column for the enrichment of OTA from beer, it was demonstrated that OTA as low as 0.51 ppb can be detected by the V-Chip system.

### 3.3. Colorimetric and Fluorometric OTA Aptasenosrs

Among various OTA detections using its aptamer, the most unique detection scheme may be the work of Yang *et al.*, in which OTA aptamer sequence was serially linked with a HRP-mimicking DNAzyme sequence as a reaction beacon [83]. For the HRP-mimicking DNAzymes, one of the G-quadruplex structures (termed as EAD2) was chosen; EAD2 sequence solely forms a unique tetrad in the presence of potassium ions and hemin by constructing a noncovalent complex of EAD2-hemin. In this scheme, the HRP activity had been initially blocked by a hairpin formation through the blocking tail next to the OTA aptamer (1.12.2). Upon interaction with OTA, the hairpin structure was loosened to form the G-quadruplex showing HRP activity with a LOD of 4 ng/mL; a similar hairpin structure, albeit with a different target molecule, had been reported as oligonucleotide-only sensor application [84].

**Figure 3:**
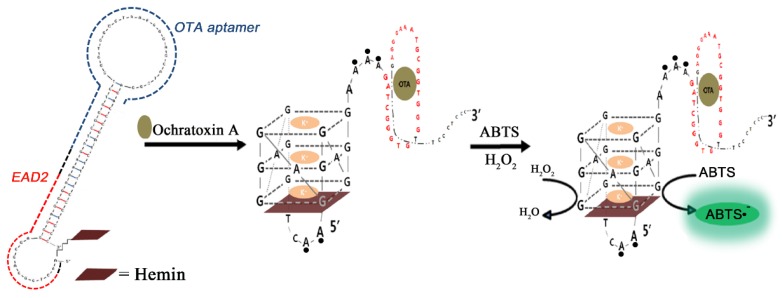
A schematic cartoon depicts a design of a hemin-conjugated DNA hairpin, and its structural change upon recognition of OTA, which forms an active G-quadruplex; the red, blue, and black dashed lines in the left denote EAD2, the OTA aptamer, and a spacer region, respectively. (Adapted from Ref. [85], Copyright 2014, Royal Society of Chemistry).

This hairpin-based detection scheme has been further refined by precisely calculating the Gibbs free energies of the hairpin formation (through the blocking tail), interaction between EAD2 and hemin, and OTA binding of the aptamer. Along with the sequence redesign, covalent attachment of hemin to EAD2 sequence enabled the hairpin-based sensor to decrease the blank signals dramatically, which could lower the LOD to the level of 1 nM (see Figure 3) [85]. As another variant of this scheme, a separate antisense oligonucleotide that plays the same role as the blocking tail was prepared, and used to hinder the HRP activity in the absence of OTA [86]. The activity of nonhybridized EAD2 (due to the aptamer binding with OTA) was linearly proportional to the content of OTA up to 12 ng/mL with an LOD of 1.6 ng/mL. Instead of using colorimetric substrate (*i.e.*, ABTS) for the HRP-mimicking DNAzyme, chemiluminescent substrate such as luminol could be employed to bring a novel path for enhancing the sensitivity [87]. For this application, a single stranded oligonucleotide was designed with EAD2 sequence at the 5′-end while OTA aptamer sequence was located at the 3′-end with a fluorescence quencher (e.g., dabsyl) at the terminus. In contrast to the previous studies, EAD2 sequence is always turn-on state as active DNAzyme regardless of OTA presence. However, the OTA binding with the portion of the 3′-end brings the quencher in close proximity to the EAD2 sequence, thereby resulting in substantial decrease in the luminol chemiluminescence (chemiluminescence resonance energy transfer, CRET). When the CRET aptasensor was applied to a coffee sample, the signal decreased with increasing OTA concentration within the range of 0.1–100 ng/mL with LOD of 0.22 ng/mL, and the recovery rate for a spiked coffee sample was estimated to be 93.3% at a concentration of 10 ng/mL [88].

Besides colorimetric detection, fluorescent transducers have been popular choices for OTA monitoring due to the long history of the fluorescent applications in the fields of genetics or protein engineering. In a typical application, OTA aptamer tagged with fluorescent dye or nanoparticles that had been initially quenched by grapheme [89], carbon nanotube (CNT) [90], or MnO_2_ nanosheet [91], increased fluorescent signals upon binding with OTA. In a similar scheme, fluorescently tagged OTA aptamers were initially hybridized with their complementary sequences with a strong quencher (*i.e.*, TARMA), minimizing the fluorescence in its resting mode. Upon binding with OTA, the fluorescent signal increased linearly in proportional to OTA concentration [92]; for corn samples, an LOD of 0.8 ng/mL was measured.

**Figure 4:**
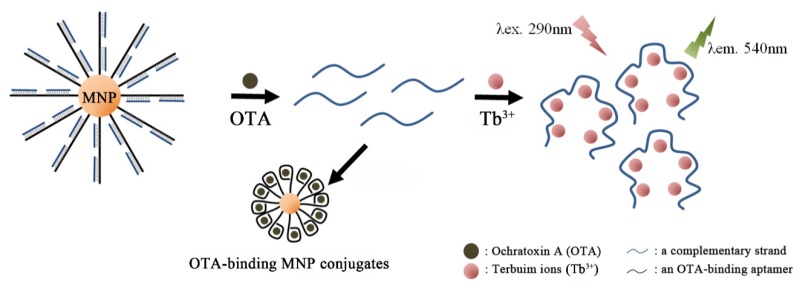
A schematic illustration shows the principle of Tb^3+^-sensitized OTA detection. In response to OTA, small complementary strands are released from magnetic nanoparticle (MNP)-aptamer nanohybrids to solution phase, which substantially enhances luminescence of Tb^3+^ ion by base coordination under exposure of UV light. (Refer to Ref. [93] for details.).

Instead of using fluorescent antisense strand toward OTA-binding aptamer, it was shown that a simple complementary strand in combination with Tb^3+^ ions could bring out a strong fluorescent signal [93]; the sensing mechanism relies on the fact that single-stranded oligonucleotides can greatly enhance the emission of Tb^3+^ ions (one of the important lanthanide ions) through energy transfer from nucleic acids to Tb^3+^, while duplexed oligonucleotides do not [94]. As shown in Figure 4, the OTA aptamer (1.12.2) was attached to streptavidin-modified magnetic beads via the avidin-biotin interaction, and was hybridized with two single stranded signal probes. In the presence of OTA in a wheat sample, OTA aptamer on a magnetic bead bound with the targets, releasing the two single stranded signal probes. Upon subsequent magnetic decantation, the signal probes in the supernatant could dramatically increase the fluorescence of Tb^3+^ ions. The Tb^3+^-based aptasensor could detect as low as 20 pg/mL of OTA with high specificity. Recently, a similar strategy was reported in which the released single stranded oligonucleotides (complementary) from the MNP-tethered OTA aptamers could scaffold the growth of silver nanoclusters (AgNCs) by addition of Ag^+^ ions with NaBH_4_, which brought out strong fluorescence (632 nm) under an excitation at 574 nm [95]. In the absence of OTA, by contrast, the same growth condition only resulted in Ag nanoparticles that emitted no fluorescence. The method exhibits superior sensitivity with an LOD as low as 2 pg/mL in the real samples of wheat.

Besides various fluorescent quenchers or enhancers, intercalators have been employed often to detect the binding of OTA aptamers with the target. As the amount of duplexed OTA aptamer would be decreased in proportion to OTA concentration, a commercially available intercalator, PicoGreen (PG), could be a fair indicator of the OTA binding [96]. The result showed that as low as 1 ng/mL of OTA could be detected with a high dynamic range of five orders of magnitude. This assay could detect a series of OTA concentrations in spiked 1% beer samples with a good tolerance toward sample matrix effects. In a similar scheme, a single stranded OTA aptamer that was forced to a hairpin structure could be also used with SYBR green I as an intercalator [97]. In this study, two novel aptamers were found after 15 rounds of *in vitro* selection, and hairpin structures with those two sequences were incorporated with SYBR Green I, which were released by the event of OTA binding. This label-free detection scheme exhibited a LOD of 9 nM, enabling linear quantification up to 100 nM.

Intriguingly, it has been shown that OTA aptamer solely can be a strongly luminescent core by employing a G-quadruplex-selective iridium(III) complex [98]. As OTA binding is accompanied by a transition from a random single-stranded DNA conformation into anti-parallel G-quadruplex structure, six Ir(III) compounds were synthesized as candidates for binding with G-quadruplex selectively, and screened by observing substantial luminescence increase upon binding with OTA. In its resting mode (in the absence of the mycotoxin), OTA aptamer (1.12.2) was hybridized with a partially complementary strand. Upon subsequent binding with OTA, a sort of G-quadruplex structure was formed, which showed strong luminescence in the presence of the Ir(III) complex; the assay exhibited linearity for OTA in the range of 0 to 60 nM and the LOD was measured to be 5 nM. Meanwhile, internally FAM-tagged OTA aptamer solely can be a unique aptasensor for the mycotoxin, which deviates in fluorescent signal by a conformation change provoked in the course of OTA binding [99]. When each tymine base of OTA aptamer (1.12.2) was systematically modified with FAM, T3- and T4-modification substantially reduced the binding affinity, suggesting that those bases are essential in OTA binding. T8-, T10-, and T19-modified OTA aptamer showed increases of FAM fluorescence on addition of OTA, while 5′-end and T30 modification decreased the signal. For instance, OTA in the range of 2–200 nM could be detected with the T30-modified aptamer, while more diluted OTA (1 nM) could be obtained with the T10-modified one.

**Figure 5:**
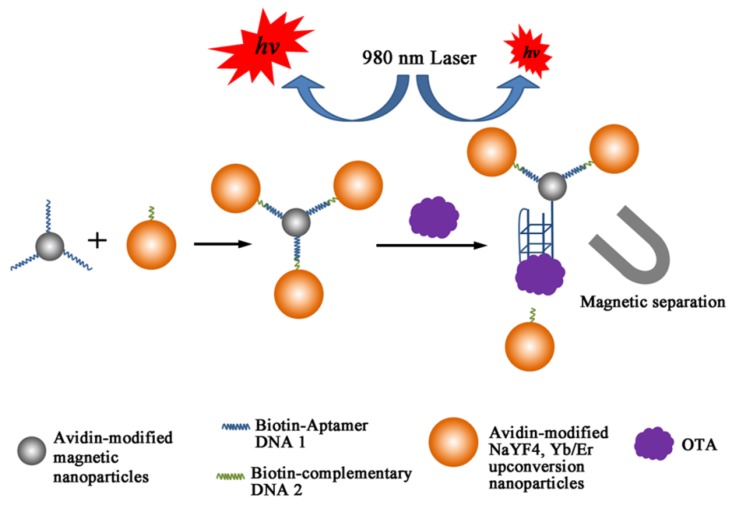
A schematic illustration of principle of OTA detection based on combining magnetic separation of aptamer-functionalized magnetic nanoparticles (MNPs) and upconversion nanoparticles (UCNPs) as luminescent labeling. (Adapted from Ref. [100], Copyright 2011, Royal Society of Chemistry).

For more sensitive detection, nanohybrids composed of MNPs and upconversion nanoparticles (UCNPs) were devised, in which OTA aptamer (1.12.2) was immobilized on MNP with the complementary strand on UCNP through biotin-avidin chemistry [100]. From the nanohybrids, UCNP was released upon binding with OTA in maize samples as shown in Figure 5, thereby reducing the upconversion fluorescence of the supernatant from magnetic decantation. Under the optimal conditions, the decreased luminescent intensity was proportional to the concentration of OTA in the range of 0.1 pg/mL to 1 ng/mL; the LOD of this scheme was measured to be 0.1 pg/mL. Furthermore, the luminescent assay was correlated with a standard ELISA method and the results confirmed that no significant deviation was found between the two methods. Instead of employing UCNPs, a similar scheme with CdTe quantum dots embedded in silica nanoparticles (QD@SiO_2_) was applied and showed a comparable sensitivity for spiked samples with a LOD of 5.4 pg/mL in a wide range of concentrations, 15 pg/mL to 100 ng/mL [101].

A similar application was devised to fabricate dual emission fluorescent aptasensor, in which green and red emitting quantum dots (gQDs and rQDs, respectively) were employed for rapid and on-site detections [102]. OTA aptamers were tagged with gQDs, while the counter strands were immobilized on AuNPs tethered on rQDs that were bigger and wrapped in silica layer. Therefore, the initial configuration where OTA aptamers (on rQDs) were hybridized with complementary strand on rQDs, only allowed the emission of red fluorescence due to fluorescence quenching by metallic AuNPs or Förster resonance energy transfer (FRET) mechanism toward rQDs. In response to OTA addition, the gQDs with OTA aptamers were liberated from rQDs and could display a distinguishable fluorescence change from red fluorescence to green in proportion to OTA concentration. The observed LOD was 1.67 pg/mL and recovery ratios over 94% were observed in spiked wine samples.

### 3.4. Electrochemical Detections with OTA Aptamers

Electrochemical methods with aptamers that have been applied to the OTA detection more or less utilize its conformation changes upon binding with the mycotoxin (G-quadruplex like) and subsequent alterations at the electrochemical interface. Upon immobilization on screen printed carbon electrodes (SPCE), OTA aptamers on the electrode could bring out sufficient electrochemical impedimetric spectroscopic (EIS) signals sufficient to monitor OTA as low as 0.15 ng/mL [103]. The increase in the R_ct_ was linearly proportional to the OTA concentration in the range of 0.15–2.5 ng/mL with recovery rates of 91%–95%. A similar but more sensitive EIS detection was achieved on the OTA aptamers tethered on AuNPs that were freshly formed on 2-aminothiophenol-functionalized reduced graphene oxide (Au-ATP-rGO) composited film on gold electrode [104]. With the composites possessing densely covered AuNPs as a versatile signal amplified platform, a label-free aptasensor was developed with a linear range of 0.1–200 ng/mL; the more OTA was captured to the electrode surface, the greater was the charge transfer resistance (R_ct_) of the electrode and the LOD was measured to be 0.03 ng/mL. In a separate impedimetric study, OTA aptamer (1.12.2) was immobilized on to iridium oxide (IrO_2_) nanoparticles, which were placed on polythionine (PTH) modified carbon electrode [105]. The EIS measurement was performed in the presence of a model redox pair ([Fe(CN)_6_]^−3/−4^) to characterize the electrochemical interfacial behavior on the IrO_2_-modified carbon electrode. It was observed that the R_ct_ increased proportionally to the OTA concentrations. The assays were performed in a range of OTA between 0.01 and 100 nM and showed one of the lowest LOD for label free impedimetric detection (14 pM or 5.7 ng/kg) with high reproducibility, good performance with white wine samples, and excellent specificity against other toxins.

Instead of being tethered on the electrode surface, OTA aptamer could be a linker strand that immobilized the AuNPs that were modified with oligonucleotides of complementary sequences [106]. In this scheme, differential pulse voltammogram (DPV) was employed to collect the signals from a redox indicator, methylene blue (MB), adsorbed on free guanine bases that were abundant in AuNPs. As OTA concentration increases in the sample solution, more AuNPs are dissociated from the electrode surface due to the loss of the linker strand, thereby lowering the redox signals of MB. An effective sensing range of 2.5 pM to 2.5 nM was received with an extremely low detection limit of 0.75 pM under optimal condition.

**Figure 6:**
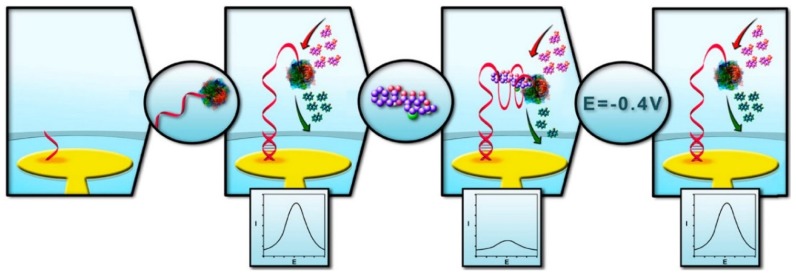
A schematic illustration of an alkaline phosphatase (ALP)-tagged OTA-binding DNA aptamer that was immobilized on a gold surface through hybridization, which undergoes a conformation switch upon OTA binding that triggers the enzyme inhibition. The enzyme activity can easily regenerated by simply applying a short potential pulse. (Adapted from Ref. [107], Copyright 2014, American Chemical Society).

Another OTA aptasensor employed electrochemically active enzymes (*i.e.*, alkaline phosphatase, ALP) as a source of electrochemical signal transduction. After self-assembling short oligonucleotides on a gold electrode, complementary to 3′-end of the aptamer sequence, ALP-aptamer conjugate were hybridized with the probe sequence leaving ALP at the 5′-terminus as shown in Figure 6 [107]. The structure switching caused by OTA binding was mainly monitored with an oxidative peak of α-naphthyl phosphate (α-NP) at 0.225 V *vs.* Ag/AgCl through DPV technique; as OTA concentration increased, the peak current decreased, as the ALP activity was substantially hindered by OTA-aptamer complexation (“signal-off”). Additionally, it was shown that OTA-aptamer complex could be easily dissociated by applying a negative pulse at −0.4 V (*vs*. Ag/AgCl) of two minute duration, thereby regenerating the electrochemical aptasensor. The ALP-based aptasensor could detect OTA as low as 2 nM in pretreated wine samples to remove polyphenol species.

In a similar variation with competitive immunoassay, ALP was employed for the signal transduction of DPV monitoring, albeit in a different way. In the proposed scheme, biotinylated OTA at a predetermined concentration competed with free OTA in sample solution [108]; as OTA concentration increases in this scheme, the amount of biotinylated OTA decreases, thereby decreasing the amount of avidin-ALP in the subsequent incubation. The cocoa samples that were pretreated with molecular imprinted polymer (MIP) columns showed a good linearity in the range of 0.15–5 ng/mL with a LOD of 0.07 ng/mL. Instead of using biotinylated OTA in a competitive assay, OTAs conjugated with cerium nanoparticles (nCeO_2_) could be a versatile indicator for the competitive assay [109]. The OTA aptamer was first immobilized on the surface of a graphene oxide (GO) modified SPCE electrode and the composite electrode was soaked with a sample solution containing both free OTA and nCeO_2_-tagged OTA in a predetermined concentration. The electrochemical signal in cyclic voltammetry was monitored by the electro-oxidation of nCeO_2_ tag in the presence of hydrogen peroxide, the catalytic substrate; according to the detection scheme, the more OTA present in the sample solution, the less the signal is observed. Under optimized conditions, the aptasensor exhibited a linear response in the range of 0.15–180 nM with an LOD of 0.1 nM for spiked corn samples.

Among various electrode materials used in OTA detection, boron-doped diamond electrode (BDD) in a single microcell appeared as an exotic material for quantitative analysis of OTA. BDD electrodes have the advantages of low background current, enhanced chemical or mechanical strengths, resistance to fouling, and a wide potential window (higher oxygen evolution potential and lower hydrogen evolution potential) [110]. In the quantification of OTA, the decrease in the square wave voltammetry (SWV) of [Fe(CN)_6_]^−4/−3^ indicator was observed without any other labeling, and showed an LOD of 0.01 ng/mL.

Another sensitive detection might be a selective electrochemiluminescent (ECL) sensor where OTA aptamer (1.12.2) is tagged with a substrate of ECL (e.g., luminol or its derivatives) as a basic signal transducer, while the counter oligonucleotide (or complementary sequence of 1.12.2) is immobilized onto AuNP-modified gold electrode [111]. The ECL-tagged OTA aptamers were initially immobilized on the gold surface through a conventional hybridization, and a decreased ECL signal was observed in the presence of OTA which binds with the ECL-tagged OTA aptamer and detaches from the active electrode. Under optimal conditions, the decreased ECL intensity was proportional to an OTA concentration in the range of 0.02–3.0 ng/mL with an LOD of 7 pg/mL. The operational sample was spiked wheat extracts and the proposed results showed good correlation with a standard method (HPLC-FD).

### 3.5. Signal Amplification Schemes with OTA Aptamers

If one intends to search for even more sensitive detection mechanisms, quantitative real-time PCR method (qRT-PCR) provides the solution for this purpose. In this scheme, OTA aptamers are initially tethered to magnetic nanoparticles in duplex form with its complementary strand. Upon binding with OTA in the sample solution, the complementary strands are dissociated with the aptamer, and eventually separate from the beads by magnetic decantation. Therefore, the presence of the complementary strand in the supernatant can be strong evidence of OTA presence, and the signal can be further amplified by conventional qRT-PCR protocols [112]. Under optimal conditions for wine samples, an LOD of 1 fg/mL was acquired in the range of 5 fg/mL to 5 ng/mL. Overall those detection methods are not only confined to OTA, but also applicable toward any small molecules with their aptamers, and are well summarized in the literature [113].

The other avenue of the amplification strategy was paved by employing rolling circle amplification (RCA), an isothermal amplification technique. RCA could be an alternative amplification route for released single strands, which avoids complicated thermal cycles and expensive instrumentations. In a representative example, the counter strands liberated from the binding interaction of OTA-aptamer constituted a circular template with an additional padlock sequence, enabling ignition of the RCA process that was monitored by the intercalating dye, SYBR Green I [114]; in this scheme, extremely sensitive detection could be facilitated for several cereal samples with an LOD of 1.2 fg/mL. In a similar application, it was demonstrated that OTA aptamers tethered on MNPs could boost the efficiency of RCA process by removing the interferences of background fluorescent noise [115]. In the absence of OTA, OTA aptamer on MNPs and complementary padlock could initiate the RCA process to bring about bright fluorescence after magnetic decantation along with the addition of QD-tagged complementary strands. However, the presence of OTA prohibited OTA aptamers (on MNPs) from binding with the padlock sequence, thereby frustrating the RCA process. The response of the optimized setup was highly linear over the wide range of 0.001–10 ppb with an LOD of 0.13 ppt. Meanwhile, instead of using intercalating fluorescent dye or QDs, long single strands produced by the RCA process could be monitored by electrochemical methods such as CV or DPV [116]. Methylene blue (MB) was adopted as the electrochemical redox probe based on the specific binding to guanine bases, after tethering the amplified strands by pre-immobilized capture strands. Under optimal conditions, ultrasensitive detection of OTA could be achieved with an LOD of 0.065 ppt.

Another isothermal amplification technique, loop mediated isothermal amplification (LAMP), can also be applied to detect the OTA content in a sample solution. In a representative study, OTA aptamers which also played a role of an inner primer among specially designed LAMP primers (two inner and two outer primers), were initially hybridized with a partially complementary strand on gold electrode [117]. The surface density of OTA aptamers on the electrode was inversely proportional to the OTA amount in the sample solution, thereby yielding a lower density of LAMP amplicons as the OTA amount increases. The lower the amplicon amount on the electrode, the higher is the ECL signal from the model indicator, Ru(phen)_3_^2+^ ions; the ECL indicator binding to the resulting LAMP amplicon cause the reduction of the ECL intensity due to the slow diffusion of Ru(phen)_3_^2+^ ions. Under optimized condition, an LOD as low as 10 fM for OTA could be observed [117].

**Figure 7:**
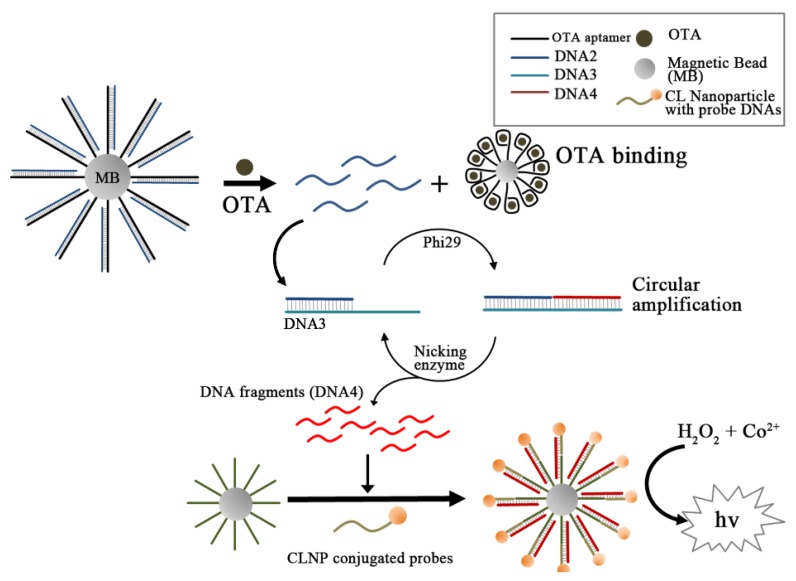
A schematic illustration of signal amplified strategy based on target-induced strand release coupling cleavage of nicking endonuclease and its application to OTA detection. In response to the formation of aptamer-OTA complex, the released DNA2 strands from magnetic beads are first hybridized with DNA3, which play a role of PCR template to produce DNA4. Since the PCR template could be recycled by the nicking enzyme, the amount of DN4 was several ten times as much as DNA2 and eventually quantitated with chemiluminescence. (Refer to Ref. [57] for details.)

In another amplifying application, the dissociated strand (complementary to OTA aptamer) hybridizes with the third strand (DNA3), which is linked at the 5′-terminus of the amplification template and can extend along the template in the presence of Phi 29 DNA polymerase (see Figure 7) [57]. Thus-formed duplex DNA is further cleaved by nicking endonuclease Nb.BbvCI and produces a short single-stranded DNA with leaving a new enzyme site for Phi 29 polymerase. The iterative process of extension and cleavage produces a much larger amount of short oligonucleotides by the Phi 29/Nb.BbvCI enzyme pair. In this study, the amount of the short oligonucleotides could be detected sensitively through an ABEI linked chemiluminescence (CL) probe. The CL intensity was linear in the range of 1.0 pg/mL to 50 ng/mL with an LOD of 0.3 pg/mL.

**Figure 8:**
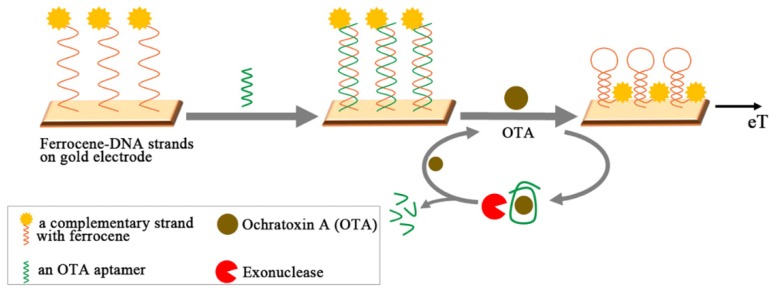
A schematic illustration shows the principle of electrochemical detection of OTA, employing exonuclease for target recycling. The complementary strands of OTA aptamer are tagged with ferrocene and form a hairpin structure, upon OTA-aptamer binding and releasing from the electrode. OTA is recycled with exonuclease dismantling the OTA aptamer. (Refer to Ref. [118] for details.)

Instead of using nicking endonuclease, a distinct amplifying mechanism was proposed that employed exonuclease [118]. As shown in Figure 8, a ferrocene-labeled and complementary oligonucleotide (S1) was immobilized onto gold electrode for the following hybridization with OTA aptamer (1.12.2). In response to OTA addition, the formation of the aptamer-OTA complex resulted in the dissociation of OTA aptamer from the duplex and departure from the gold electrode. Subsequently, the remaining S1 chain was forced to form a hairpin structure, yielding the dramatic increase of the faradaic current of the ferrocene label. At the same time, once liberated aptamer-OTA complex was further dismantled by exonuclease thereby releasing an intact OTA molecule, which eventually enabled the target to be recycled. Overall, the recycling of OTA was accompanied by amplified surface hairpin formation (*i.e.*, ten-fold), which led to an increase in the faradaic current for OTA in the observations from differential pulse voltammetry (DPV). Based on this strategy, the ultrasensitive aptasensor for the detection OTA in wheat flour demonstrated wider linear range (5 pg to 10 ng/mL) with a low LOD of 1.0 pg/mL. In addition, the acquired data showed good correlation with ELISA using a commercialized detection kit. The same concept of “target recycling” could be found in the fluorescent detection scheme employing fluorescein labeled OTA aptamer and graphite nanoparticles [119]. Initially quenched fluorescence of the aptamer with graphite nanoparticles could be revived upon binding with the target by releasing the aptamer-OTA complex. Furthermore, DNase I decomposed the aptamer-OTA complex, thereby amplifying the fluorescent signal via recycling the once liberated OTA molecules. The similar augmentation of the signal could be observed in ECL of CdTe nanoparticles on a gold electrode [120]. In the absence of OTA, the hybridized OTA aptamer with its complementary strand tethered on gold electrode played a role in blocking or retarding the ECL process of CdTe nanoparticles, while the presence of OTA in the sample solution released the OTA-aptamer complex. RecJ exonuclease decomposed not only the oligonucleotides of OTA-aptamer complex but also the remaining counter strands on the electrode, facilitating the recycling of OTA. Due to the exonuclease-catalyzed target recycling amplification, the inhibition effect of CdTe ECL is significantly enhanced to achieve an LOD of 0.64 pg/mL.

**Figure 9:**
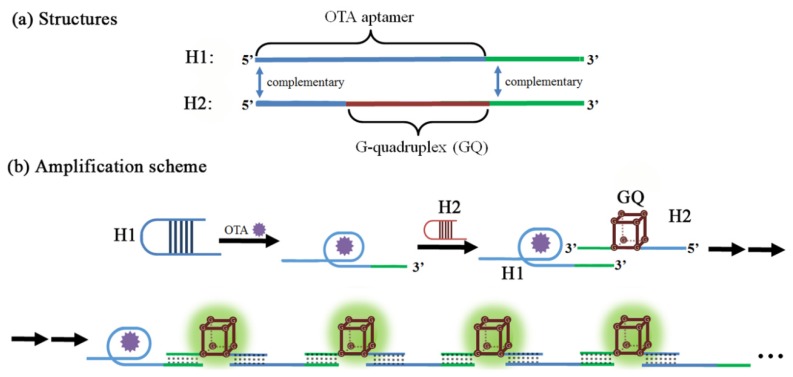
A schematic cartoon showing OTA-induced hybridization chain reaction (HCR) process and the detection mechanism. (Refer to Ref. [121] for details.) Conceptual structure of the oligonucleotide H1 and H2 (**a**), and the subsequent chain elongation mechanism (**b**).

Another exotic amplifying mechanism is related to the hybridization chain reaction (HCR), which is performed without any of the enzyme such as polymerase or nucleases which were major players in the previous amplification schemes [121]. As shown in Figure 9, two hairpin oligonucleotides (H1 and H2) were designed to form an amplified aptasensor; OTA aptamer is located in the 5′ end of H1 while G-quadruplex is at the center of H2. Meanwhile, the 3′ end of H1 is complementary to the 3′ end of H2 while the 5′ end of H2 is the complement of the 5′ end of H1. Upon OTA binding of H1 strand, the hairpin structure of H1 is opened to expose the 3′ end of H1, which will hybridize with the 3′ end of H2 to form enzymatic G-quadruplex with the 5′ end tail opened. In this way, each copy of OTA can propagate a chain reaction of hybridization between H1 and H2 strands, which contains an HRP-mimicking G-quadruplex unit in each cycle. As such, the presence of OTA generates many copies of HRP-mimics and produces a strong colorimetric signal correlated with the concentration of OTA in a range of 0.01–0.32 nM with an LOD of 0.01 nM under optimal conditions.

In another application using HCR construct, the OTA aptamer and G-quadruplex sequence was initially built on to form a supersandwich nanostructure, where both sequences were integrated into the nanostructure on a gold electrode; in this superstructure, G-quadruplexes were in their full working forms with the addition of hemin [122]. In the absence of OTA, the nanostructures were designed to quench ECL transduction (of O_2_/S_2_O_8_^2−^ pair) by depleting molecular O_2_ in advance by HRP activity of G-quadruplex. However, the addition of OTA and the subsequent formation of OTA-aptamer complex gradually dismantled the supersandwich into monomeric hairpins, which diffused out to the solution phase. Due to the destruction of the blocking supersandwich layer on a gold electrode, the ECL signal increased proportionally to the concentration of OTA. Furthermore, the concomitant addition of exonuclease, RecJ, continuously decomposed the OTA aptamer in the solution phase, thereby liberating OTA molecules and enabling OTA to be recycled. Due to the OTA recycling, a minute amount of OTA could be detected with an LOD of 75 fg/mL.

## 4. OTA Detection Methods Related to Their Physicochemical Characteristics

**Figure 10:**
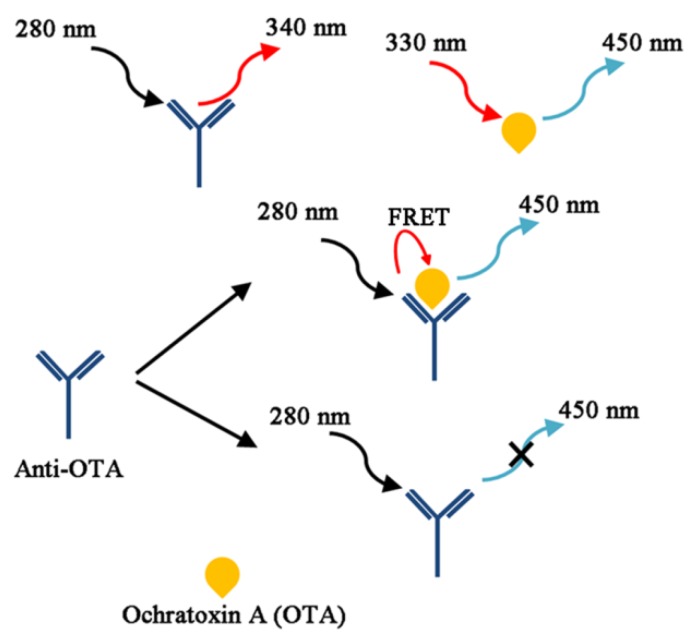
A schematic representation showing a label-free, direct, and noncompetitive homogeneous Förster resonance energy transfer (FRET) immunoassay system for detection of quantitative analysis of OTA, based on the intrinsic fluorescence properties of the anti-OTA and OTA complex (Adapted from Ref. [123], Copyright 2011, Royal Society of Chemistry).

From the chemistry viewpoint, OTA is a compound produced by linking phenylalanine with dihydroisocoumarin through an amide bond as drawn in Figure 1, which defines the whole physicochemical properties of OTA. In the same line, the toxicity of OTA is conjectured to be related to its interference with the metabolism of phenylalanine. On the other hand, the chemistry of OTA may provide unique solutions for its detection, such as taking advantage of its intrinsic fluorescent characteristics. It is known that a weakly acidic solution of OTA displays a maximum emission wavelength at 450 nm when excited at 330 nm, while tryptophan residues in proteins usually have intrinsic fluorescence, emitting light at 340 nm at the excitation of 280 nm. Since the excitation/emission characteristics of OTA are serendipitously well matched with protein fluorescence to construct a FRET pair for OTA detection, a novel protocol could be devised without any fluorescent label as seen in Figure 10. Li, *et al.* in 2011 reported that OTA and its antibody can be employed to directly detect the presence of the mycotoxin by simply observing the emission at 450 nm in the presence of OTA upon excitation at 280 nm [123]; no emission was observed in the absence of OTA. Under optimal buffer condition, the recovery rate reached 100% in a spiked wheat sample of OTA (20 ng/mL OTA), and an LOD of 1 ng/mL was measured.

In their extended study, similar fluorescent immunoassay was attempted, but monitoring excitation wavelength changes at a fixed emission wavelength of 450 nm as OTA binds with its antibody [124]. Based on the observation that OTA exists in a dianionic form when captured with its antibody, the maximum excitation wavelength (λ_ex_) was changed from 340 nm to 380 nm, even in slightly acidic condition at the given emission wavelength (λ_em_ = 450 nm); in alkaline media (when the dianionic form is prevailing), it is already known that the maximum excitation is 380 nm irrespective of antibody binding. Intriguingly, this observation becomes important because the previous FRET assay is inefficient for determining the OTA concentrations in polyphenol-rich beverages (*i.e.*, wines); polyphenolic compounds exhibit strong fluorescence at 450 nm with a substantially high quantum yield upon UV excitation [123,125]. For the detection of OTA concentration in polyphenol-rich red wine, white wine, and grape juice, the LODs were measured to be 2.1, 1.2, and 2.0 ng/mL, respectively.

Recalling that OTA is structurally composed of phenylalanine that is linked with dihydroisocoumarin via an amide bond, another interesting strategy was devised employing carboxypeptidase (CPY) and thermolysin (TLN) that are peptidases with relatively broad substrate specificity which can hydrolyze OTA molecules [126]. The conductometric detection scheme used in this paper could monitor changes in surface polarization or electrical double layer structures during the hydrolysis of the *pseudo*-dipeptide (OTA). One pair of gold interdigitated electrodes was first fabricated to support those peptidases, and CPY and TLN on the electrodes was crosslinked by glutaldehyde vapor in presence of lysine-rich BSA protein as buffering cushion, which made a robust and flexible sensing film. The enzyme modified electrodes were immersed in buffer solution (5 mM phosphate buffer of pH 7) and an alternating voltage (10 mV amplitude at 100 kHz) was applied to observe non-faradaic responses of double layer charging, or polarization changes in the microelectrodes. In a range of OTA concentrations (1–75 μM), the electrochemical biosensor demonstrated good reproducibility with fair LODs (1 μM for CPY and 0.7 μM for TLN, respectively).

Raman spectroscopy is another invaluable tool due to its advantages in detecting mycotoxins in grains and oil seeds [127,128,129]. Compared to other spectroscopic methods (e.g., FTIR) in mycotoxin assays, Raman spectroscopy can provide well resolved bands of an individual mycotoxin and allow the acquisition of spectra in aqueous solution. Nonetheless, direct observation of OTA molecules by Raman spectroscopy was relatively rare except for several studies for aflatoxins [130]. Due to the small cross section of Raman scattering, in most detection schemes, SERS (surface enhanced Raman spectroscopy) has been a monitoring tool for diverse additives or toxins in the chemical analysis of food [131]. Even for the SERS assay of aflatoxin, the LODs of directly adsorbed aflatoxin were measured to be in the range of 13–36 ng/g, which is relatively higher than those immunoassays or aptamer-based assays described above [130]. Since a typical SERS enhancement factor reported in previous studies was about an order of 10^7^, or up to 10^14^ at extremely high enhancement, the preparation of SERS susceptible surface is a linchpin factor for the sensitive detection of small molecules. Therefore, it appears that the fabrication of reproducible enhancing surfaces and the following SERS detection toward OTA will be relentlessly attempted to harness the advantages of SERS. Meanwhile, instead of detecting vibrational fingerprints of OTA directly, changes of SERS signal of adsorbed OTA aptamers (Cy5-oligonucleotides) on silver nanoparticles were pursued as an alternative approach [132]. In this study, SERS signal of Cy5 is a tracking marker for OTA contents; as OTA concentration increases, the SERS signal proportionally decreases due to the formation of stable OTA-aptamer complexes, which are released from the nanoparticle surface. OTA concentrations in the range of 0.1–10 nM were determined with an LOD of 0.1 nM (0.07 ng/g). In another detection of OTA using nanostructured SERS surface, OTA molecules are captured by specific OTA aptamer on the SERS active surface placed in the microfluidic channel [133]. In this study, metallic nanostructures for SERS detection were fabricated with electron-beam lithography on a glass coverslip and the vibrational spectra of OTA were directly observed, albeit with the aid of the aptamer. For the multiplexed SERS detection of OTA and aflatoxins B1 (AFB1), each aptamer was immobilized on Ag@Au core-shell nanoparticles (CS-NPs) bearing distinct SERS labels such as 4-nitrophenol (4-NTP) and 4-aminophenol (4-ATP), respectively [134]. Those two CS-NPs were initially hybridized with magnetic nanoparticles bearing their complementary strands so that a single MNP could embrace both nanoparticles. Upon binding with targets, the corresponding CS-NPs would be released in proportion to the amount of targets after magnetic decantation. In this scheme, SERS tags engineered Raman aptasensors could be developed for the double detection of the dual mycotoxins with LODs of 0.006 ng/mL for OTA and 0.03 ng/mL for AFB_1_.

**Figure 11:**
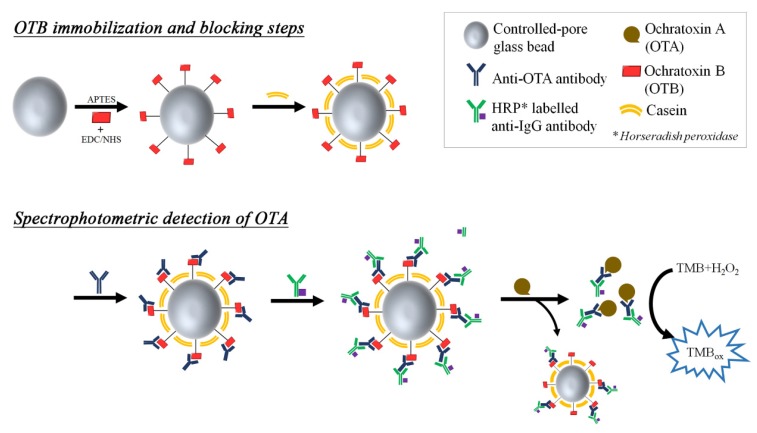
A schematic cartoon showing steps in the construction of the OTB based biosensing glass bead material and the displacement reaction upon incubation with OTA. (Refer to Ref. [137] for details.)

Ochratoxin B (OTB) is the non-chlorinated analogue of OTA, produced in a 1:5 ratio by the same fungi strands (aspergillus and penicillium) as in OTA production [135]. Although the related studies are rare due to its mild toxicity, OTB is usually employed as an interfering analyte in the immunoassays for OTA detection [89], and as an internal standard in LC-MS detection of OTA [136]. In a separate displacement immunoassay (see Figure 11), OTB was immobilized onto silica nanoparticles and OTA antibodies were attached by weak interactions between OTB and OTA-antibodies [137]; the OTA antibodies were further labeled with secondary anti-IgG antibodies containing HRP, as a colorimetric/fluorometric beacon (see Figure 10). Upon addition of OTA, antibodies that had initially associated with OTB were released to bind with OTA in the solution phase which has a stronger binding affinity for the antibodies. Therefore, the HRP signals in the supernatant after centrifugation were proportional to the amount of OTA. In regard to the detection of small molecules such as mycotoxins, the competitive immunoassay has been the most popular format compared to direct immunoassay, so that OTB appears to be positioned as an essential OTA surrogate in the many competitive detection schemes.

## 5. Conclusions

In this review, a broad range of OTA detection schemes have been briefly surveyed, which encompass conventional chromatographic methods, immunoassays, aptamer-related techniques, and miscellaneous OTA-specific assays together with some details on working principles and detection limits. Although the toxicity of aflatoxins are overwhelming compared to ochratoxins or other mycotoxins, ochratoxins appear to have the largest volume of research papers (or professional investigations). This may be caused partly by the extreme toxicity of aflatoxins that hampers extensive and wide studies in laboratories worldwide. Nonetheless, it seems clear that the huge network of experimental results on ochratoxins garnered hitherto can also provide deeper insight into other “mycotoxin detections”, which are emerging as a new challenge towards the public health systems in the developed countries. Summarizing the recent studies on OTA, aptamer related detection schemes have widened their scope largely due to their chemical stability, high sensitivities, and broad applicability to other functional materials like nanoparticles or other nanomaterials. Moreover, recent explosive advances on functional nanomaterials such as metallic/oxide nanoparticles, graphenes, luminescent nanoparticles, supramolecules, and nanobio-hybrids could well boost pioneering efforts for the advanced and smart detection modes on the mycotoxin.
